# A Chimera Na^+^-Pump Rhodopsin as an Effective Optogenetic Silencer

**DOI:** 10.1371/journal.pone.0166820

**Published:** 2016-11-18

**Authors:** Mohammad Razuanul Hoque, Toru Ishizuka, Keiichi Inoue, Rei Abe-Yoshizumi, Hiroyuki Igarashi, Takaaki Mishima, Hideki Kandori, Hiromu Yawo

**Affiliations:** 1 Department of Developmental Biology and Neuroscience, Tohoku University Graduate School of Life Sciences, Sendai, 980–8577, Japan; 2 Department of Life Science and Applied Chemistry, Nagoya Institute of Technology, Showa-ku, Nagoya, 466–8555, Japan; 3 OptoBioTechnology Research Center, Nagoya Institute of Technology, Showa-ku, Nagoya, 466–8555, Japan; 4 PRESTO, Japan Science and Technology Agency, 4-1-8 Honcho, Kawaguchi, Saitama, 332–0012, Japan; 5 Frontier Research Institute for Material Science, Nagoya Institute of Technology, Showa-ku, Nagoya, 466–8555, Japan; 6 Department of Physiology and Pharmacology, Center for Neuroscience, Tohoku University Graduate School of Medicine, Sendai, 980–8577, Japan; 7 Tohoku University Division For Interdisciplinary Advanced Research and Education, Sendai, 980–8578, Japan; Dalhousie University, CANADA

## Abstract

With the progress of optogenetics, the activities of genetically identified neurons can be optically silenced to determine whether the neurons in question are necessary for the network performance of the behavioral expression. This logical induction is expected to be improved by the application of the Na^+^ pump rhodopsins (NaRs), which hyperpolarize the membrane potential with negligible influence on the ionic/pH balance. Here, we made several chimeric NaRs between two NaRs, KR2 and *Ia*NaR from *Krokinobacter eikastus* and *Indibacter alkaliphilus*, respectively. We found that one of these chimeras, named *I*_1_*K*_6_NaR, exhibited some improvements in the membrane targeting and photocurrent properties over native NaRs. The *I*_1_*K*_6_NaR-expressing cortical neurons were stably silenced by green light irradiation for a certain long duration. With its rapid kinetics and voltage dependency, the photoactivation of *I*_1_*K*_6_NaR would specifically counteract the generation of action potentials with less hyperpolarization of the neuronal membrane potential than KR2.

## Introduction

A neural system is a kind of computing system comprised by a vast number of neurons. Understanding of the significance of a neuron or a set of neurons in a network has been one of the major goals of neuroscience. In recent years the optical methods have been developed to manipulate genetically targeted neurons using light under precise spatiotemporal resolution [1–4]. For example, the neuronal excitability is enhanced by light if light-sensitive cation channels such as channelrhodopsin-2 (ChR2) are expressed in a neuron [5,6]. The resulting neural network or behavior responses suggest that the stimulated neuron is involved in the responses as a sufficient condition, although it is necessary to avoid oversimplification [4]. On the other hand, optical silencing of the neural activity could determine whether the neuron in question is necessary for the network performance of the behavioral expression.

In the field of the optogenetics, the microbial Cl^-^-pump rhodopsins such as a halorhodopsin from *Natronomonas pharaonis* (NpHR) and the microbial H^+^-pump rhodopsins such as archaerhodopsins from *Halorubrum sodomense* (AR3/ArchT) are now the most widely used loss-of-function tools, which effectively hyperpolarize the membrane potential to inhibit the generation of action potentials [7–11]. Recently, Cl^-^/anion channel rhodopsins have become another potential candidates for the purpose of optogenetic silencing of the neural activities [12–15]. Although they have made great progress, these tools still have some disadvantages such as inevitable changes in the ionic/pH balance between the intracellular and extracellular milieu [1,16,17]. In addition, in the case of Cl^-^/anion channel rhodopsins, the direction of the membrane potential change should be dependent on the Cl^-^-equilibrium potential, which can be affected by many factors such as development, localization and disease [18–25]. On the other hand, a new microbial rhodopsin named KR2 from the marine flavobacterium *Krokinobacter eikastus*, was characterized as one of the light-driven Na^+^ pumps that transport Na^+^ from the inside to the outside of the expressed cell under physiological conditions [26]. As the natural excitatory signals generally induce Na^+^ influx through non-selective cation channels, the Na^+^ pump rhodopsins (NaRs) are ideal tools to reduce the neuronal excitability by light, but with minimal influence on the ionic/pH balance [27].

Recently the numbers of NaRs are increasing to form a large subfamily of microbial rhodopsins [28]. Here, we characterized one of the microbial rhodopsins named *Ia*NaR from *Indibacter alkaliphilus* as a new NaR that transports Na^+^ from the inside to the outside of the expressed cell under physiological conditions. The ion-transporting activity of *Ia*NaR was inefficient when exogenously expressed in mammalian cells. However, one of the chimeric NaRs between *Ia*NaR and KR2 exhibited some improvements in the membrane targeting and photocurrent properties over native ones. It is anticipated that the chimeric NaRs will become useful to counteract the generation of action potentials when expressed in neurons as an optogenetic silencing tool.

## Materials and Methods

### Protein expression and purification

The synthesized *Ia*NaR gene, whose codons were optimized for *E*. *coli* expression, was inserted into the Nde I-Xho I site of pET21a vector, with the resulting construct encoding a His_6_ tag at the C-terminus. The expression plasmid of *Ia*NaR was transformed in to *E*. *coli* C41(DE3) and the rhodopsins were overexpressed in the cells induced with 1 mM isopropyl-β-D-thiogalactopyranoside (IPTG) and 10 μM all-*trans* retinal for 4 hours. The crude membrane was solubilized with 1.5% n-dodecyl-β-D-maltoside (DDM), and the solubilized fraction was purified by TALON Metal Affinity Resin (Clontech Laboratories Inc., Mountain View, CA, USA).

### Measurement of ion-transport activity

The *Ia*NaR expressing cells were collected by centrifugation (4,800×*g*, 2 min), washed three times and resuspended in the same solvent (in mM), 100 NaCl, 100 Na_2_SO_4_ or 100 KCl, as for ion-transport measurement. The cell suspension was placed in the dark and then illuminated by using a 1-kW tungsten-halogen projector lamp (Master HILUX-HR, Rikagaku, Japan) through a glass filter (Y-52, AGC Techno Glass, Japan) for 2.5 min at wavelengths > 500 nm. The light-induced pH changes during incubation were monitored with a pH meter (F-55, Horiba, Japan).

### Molecular biology

**Fig 1:**
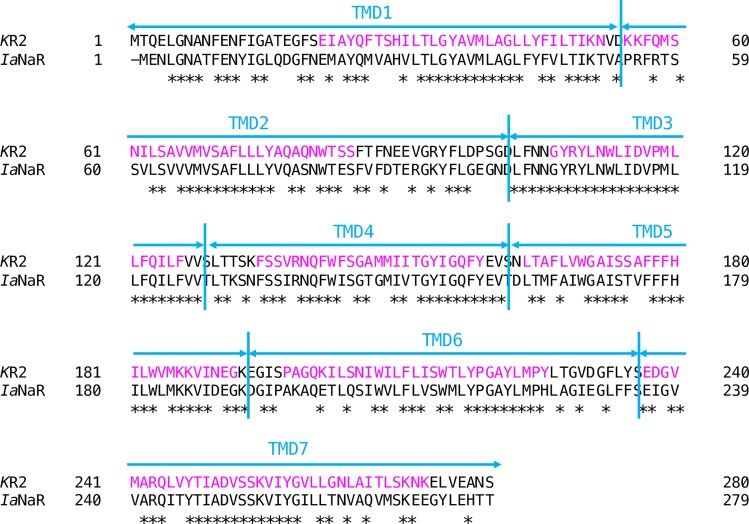
Primary structure of the Na^+^ pump rhodopsin (NaR) apoproteins. The sequence alignment of two NaRs, one from *Krokinobacter eikastus* (KR2, amino acids 1–280) and the other from *Indibacter alkaliphilus* (*Ia*NaR, amino acids 1–279) is shown. Identical amino acids are indicated by an asterisk. The seven transmembrane helixes of KR2 (Kato et al., 2015) are lettered in magenta. The amino acid sequences of the apoprotein were divided into seven transmembrane domains (TMDs) so that each TMD contain a transmembrane helix (cyan vertical lines). These segments are referred to (from the N-terminal to C-terminal) as “TMD1”, “TMD2”, “TMD3”, “TMD4”, “TMD5”, “TMD6”, and “TMD7”, respectively.

The CMV promoter-based expression plasmids encoding KR2, *Ia*NaR, or its chimeras fused in-frame with a membrane trafficking signal (TS), an enhanced yellow fluorescent protein (eYFP), and an ER export signal (ER_ex_) were prepared as previously reported [27]. Both TS and ER_ex_ were derived from a Kir2.1 potassium channel with amino acid sequences of “*N-*KSRITSEGEYIPLDQIDINV-*C* (20 amino acids)” and “*N-*FCYENEV-COOH (7 amino acids)”, respectively [29]. The cDNAs encoding chimeras between E. coli codon-optimized *Ia*NaR and human codon-optimized KR2 were prepared using In-Fusion cloning technology (Takara Bio, Shiga, Japan). Briefly, the amino acid sequences of the apoprotein were divided into seven transmembrane domains (TMDs) so that each TMD contained a transmembrane helix. These segments are referred to (from N-terminal to C-terminal) as “TMD1”, “TMD2”, “TMD3”, “TMD4”, “TMD5”, “TMD6”, and “TMD7” (Fig 1). The N-terminal transmembrane domains (TMDs) of KR2 were replaced in order with the homologous counterparts of *Ia*NaR and thus we prepared 6 chimeras; *I*_1_*K*_6_NaR from TM1 (Met^1^-Val^52^) of *Ia*NaR and TM2-7 (Asp^54^-Ser^280^) of KR2, *I*_2_*K*_5_NaR from TM1-2 (Met^1^-Asn^100^) of *Ia*NaR and TM3-7 (Asp^102^-Ser^280^) of KR2, *I*_3_*K*_4_NaR from TM1-3 (Met^1^-Val^127^) of *Ia*NaR and TM4-7 (Ser^129^-Ser^280^) of KR2, *I*_4_*K*_3_NaR from TM1-4 (Met^1^-Val^160^) of *Ia*NaR and TM5-7 (Ser^162^-Ser^280^) of KR2, *I*_5_*K*_2_NaR from TM1-5 (Met^1^-Lys^192^) of *Ia*NaR and TM6-7 (Glu^194^-Ser^280^) of KR2 and *I*_6_*K*_1_NaR from TM1-6 (Met^1^-Phe^234^) of *Ia*NaR and TM7 (Ser^236^-Ser^280^) of KR2. For optimal expression in cultured neurons, the CMV enhancer/promoter was replaced with the CaMKIIα promoter. All constructs were verified by sequencing.

All constructs were verified by sequencing, and submitted to DDBJ/EMBL/GenBank (http://www.ddbj.nig.ac.jp/index-e.html) with accession codes in the parentheses; human codon-optimized KR2 (Acc# LC012789), E. coli codon-optimized *Ia*NaR (Acc# LC187664), *I*_1_*K*_6_NaR (Acc# LC187665), *I*_2_*K*_5_NaR (Acc# LC187666), *I*_3_*K*_4_NaR (Acc# LC187667), *I*_4_*K*_3_NaR (Acc# LC187668), *I*_5_*K*_2_NaR (Acc# LC187669) and *I*_6_*K*_1_NaR (Acc# LC187670),

### Mammalian cell culture

The electrophysiological assays of NaRs were made using ND7/23 cells as described previously [30]. Briefly, the cells were grown in Dulbecco’s modified Eagle’s medium (Wako, Osaka, Japan) supplemented with 2.5 μM all-*tran*s retinal, 10% fetal bovine serum under a 5% CO_2_ atmosphere at 37°C. The expression plasmid encoding KR2, *Ia*NaR, or its chimeras was transiently transfected in ND7/23 cells using Effectene Transfection Reagent (Qiagen, Tokyo, Japan) according to the manufacturer’s instructions. Electrophysiological recordings were then conducted 20–48 h after the transfection. Successfully transfected cells were identified by the presence of eYFP fluorescence.

Under anesthesia with a mixture (1 ml/kgBW) of ketamine (50 mg/ml, Daiichi Sankyo Co. Ltd., Tokyo, Japan) and xylazine (xylazine hydrochloride, 10 mg/ml, Sigma-Aldrich, St. Louis, MO, USA), the cortical neurons were isolated from embryonic day 16 Wistar rats (Japan SLC Inc., Shizuoka, Japan), using Nerve-Cells Dispersion Solutions (Wako) according to the manufacturer’s instructions, and were grown in neuronal culture medium (Wako) under a 5% CO_2_ atmosphere at 37°C. The neuronal expression plasmids were transiently transfected into cortical neurons by calcium phosphate transfection at days *in vitro* (DIV) 5 or 6. Electrophysiological recordings were then conducted at DIV 22–25 with neurons identified as expressing eYFP fluorescence by a conventional epifluorescence system.

All animal experiments were approved by the Tohoku University Committee for Animal Experiments (Approval No. 2014LsA-001) and were carried out in accordance with the Guidelines for Animal Experiments and Related Activities of Tohoku University as well as the guiding principles of the Physiological Society of Japan and the National institutes of health (NIH), USA. The number of animals in this study was kept to a minimum and, when possible, all animals were ketamine- xylazine anesthetized to minimize suffering.

### Electrophysiology

All experiments were carried out at room temperature (23 ± 2°C). Photocurrents were recorded as previously described [30] using an EPC-8 amplifier (HEKA Electronic, Lambrecht, Germany) under a whole-cell patch clamp configuration. The data were filtered at 1 kHz and sampled at 10 kHz (Digidata1440 A/D, Molecular Devices Co., Sunnyvale, CA) and stored in a computer (pClamp10.3, Molecular Devices). The photocurrent peak and steady-state (at the end of a 1-s light pulse) amplitudes were expressed as effective values (*I*_p_ and *I*_ss_, respectively) after being divided by the whole-cell capacitance, which is proportional to the cell’s surface area. For evaluating the magnitude of inactivation, the difference between *I*_p_ and *I*_ss_ was divided by *I*_p_.

The internal pipette solutions for the whole-cell voltage clamp recordings from the ND7/23 cells contained (in mM): 20 NaOH, 100 L-glutamic acid Na salt, 5 EGTA, 50 HEPES, 2.5 MgCl_2_, 2.5 MgATP, 0.1 leupeptin and pH 7.3 (adjusted by HCl). The cells were continuously superfused at a rate of 2 mL/min by ACSF solution (in mM): 125 NaCl, 2.5 KCl, 25 NaHCO_3_, 1.25 NaH_2_PO_4_, 2 CaCl_2_, 1 MgCl_2_, 11 glucose, pH 7.4 and equilibrated with 95% O_2_ and 5% CO_2_.

The internal pipette solution for the whole-cell current-clamp recordings from cortical neurons contained (in mM): 125 K-gluconate, 10 NaCl, 0.2 EGTA, 10 HEPES, 1 MgCl_2_, 3 MgATP, 0.3 Na_2_GTP, 10 Na_2_-phosphocreatine, 0.1 leupeptin and pH 7.4 adjusted with KOH. The cells were continuously superfused at a rate of 2 mL/min by ACSF solution equilibrated with 95% O_2_ and 5% CO_2_. In all cortical neuron experiments, the ACSF contained 100 μM picrotoxin (Nacalai Tesque, Kyoto, Japan) and 1 mM kynurenic acid (Sigma-Aldrich Co. LLC, St Louis, MS., USA) to block all synaptic inputs. The directly measured liquid junction potential was 11.4 mV and was compensated for.

### Optics

Irradiation was performed using a SpectraX light engine (wavelength > 90% of the maximum, 534–600 nm, Lumencor Inc., Beaverton, Oregon, USA) controlled by computer software (pCLAMP10.3, Molecular Devices). The power density (irradiance) of the light was directly measured under microscopy by a visible light-sensing thermopile (MIR-101Q, SSC Co., Ltd., Kuwana City, Japan) and was 99 mW/mm^2^. To investigate the action spectrum, irradiation was made at wavelengths (nm, >90% of the maximum, irradiance): 390 ± 18 (3.4 mW/mm^2^), 438 ± 24 (3.0 mW/mm^2^), 475 ± 28 (2.7 mW/mm^2^), 513 ± 17 (2.7 mW/mm^2^), 549 ± 15 (2.6 mW/mm^2^), 575 ± 25 (2.8 mW/mm^2^) and 632 ± 22 (2.8 mW/mm^2^).

### Cytochemistry

Live cultured cells were reacted with 2 μg/ml of wheat germ agglutinin (WGA) conjugated with Alexa Fluor 633 (W21404, Thermo Fisher Scientific, Waltham, MA, USA) for 10 min at 37°C, then fixed with 4% paraformaldehyde. Localization was assessed by confocal microscopy (LSM710, Carl Zeiss, Oberkochen, Germany) equipped with ×63 oil objective lens. For the detection of each fluorescence substrate, the following optical combinations were used: a 488 nm argon laser and 523–620 nm bandpass filter for eYFP, a 633 nm HeNe laser and 638–747 nm bandpass filter for WGA.

For images of primary cultured cortical neurons, each pixel value of the relative fluorescent intensity was profiled along horizontal/vertical axes, which were delineated to pass the centroid of the cell (Zen software, Carl Zeiss), and the cross-correlation analysis between eYFP fluorescence and WGA florescence (Alexa Fluor 633) was performed for the pixel values between two points that were outside of the membrane to reach half of the peak amplitude of the WGA fluorescence (Clampfit 10, Molecular Devices). The overlapping index of a cell was defined as the average of 14 cross-correlation coefficients calculated for each lag of 0, ±1, ±2 and ±3 (the maximal distance, ±0.4 μm) along the horizontal and vertical axes. It was expected to be +1.0 when both profiles between eYFP and WGA are completely overlapped and to be -1.0 when there was no overlap.

### Statistical analysis

All data in the text, figures and tables are expressed as mean ± SEM and were evaluated with the Mann-Whitney *U*-test for statistical significance unless otherwise noted. It was judged as statistically insignificant when P > 0.05.

## Results

### Characterization of *Ia*NaR

Fig 1 shows the sequence of *Ia*NaR aligned with the sequences of KR2, another NaR from marine flavobacterium. They were 86% homologous and consisted of 7 domains with each transmembrane helix (TMD1-7).

**Fig 2:**
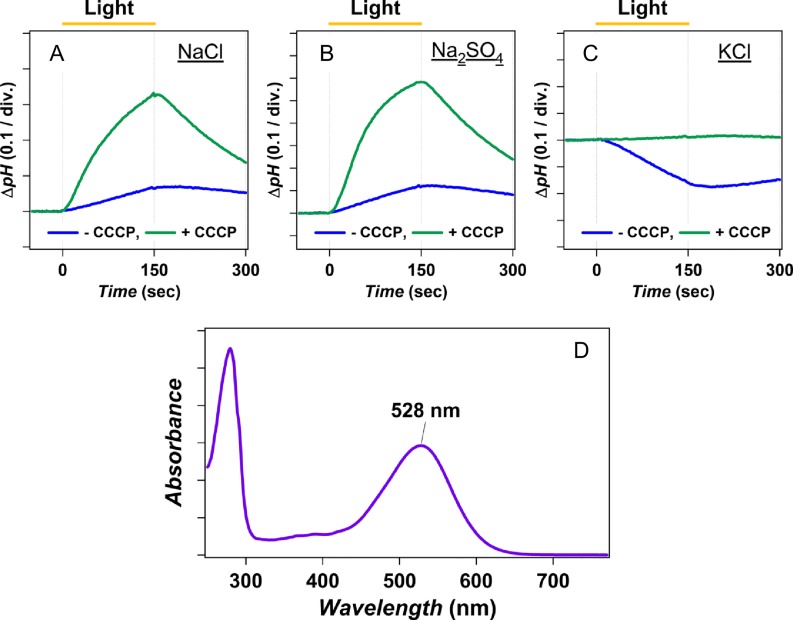
Ion-transport activity of *Ia*NaR. (**A**-**C**) Light-induced pH changes upon light-illumination (>500 nm, indicated by yellow lines) of the *E*. *coli* cells expressing *Ia*NaR in 100 mM NaCl (**A**), 100 mM Na_2_SO_4_ (**B**) and 100 mM KCl (**C**) without (blue lines) and with CCCP (green lines). (**D**) The UV-visible absorption spectrum of purified *Ia*NaR.

To study the ion-transport selectivity of *Ia*NaR, the protein was expressed in *E*. *coli* cells and the light-induced pH change was monitored upon illumination of the cell suspension. Fig 2A shows the light-induced pH change of the cell suspension in 100 mM NaCl. We observed an increase in pH (Fig 2A, blue line) that was enhanced by the addition of carbonylcyanide-m-chlorophenylhydrazone (CCCP) (Fig 2A, green line). This result indicates that the increase in pH represents the secondary H^+^-uptake into the *E*. *coli* cell body to compensate the hyperpolarized membrane potential generated by the transport of ions other than H^+^ by *Ia*NaR. In order to reveal the type of ion transported by *Ia*NaR, we investigated the cation and anion dependencies. In 100 mM Na_2_SO_4_, an increase in the pH identical to the result with NaCl was observed (Fig 2B). Thus, the transport of *Ia*NaR does not depend on the type of anion in the suspension. By contrast, a decrease in pH was observed in the suspension containing 100 mM KCl (Fig 2C). Because it diminished with the addition of CCCP (Fig 2C, green line), this result shows that *Ia*NaR pumps H^+^ in the presence of larger cations. This cation dependence of *Ia*NaR is identical to KR2 [26] suggesting that *Ia*NaR is a light-driven Na^+^ pump as expected from its amino-acid sequence including the characteristic NDQ-motif which is composed of Asn^111^, Asp^115^ and Gln^122^ for *Ia*NaR [26].

To obtain the absorption spectrum of *Ia*NaR, we purified the protein expressed by *E*. *coli* and solubilized in n-dodecyl-β-D-maltoside (DDM). The spectrum of solubilized *Ia*NaR (Fig 2D) showed its absorption maximum at λ_max_ = 528 nm, which is close to that of KR2 (λ_max_ = 524 nm) [26] suggesting that their protein structures should be identical.

**Fig 3:**
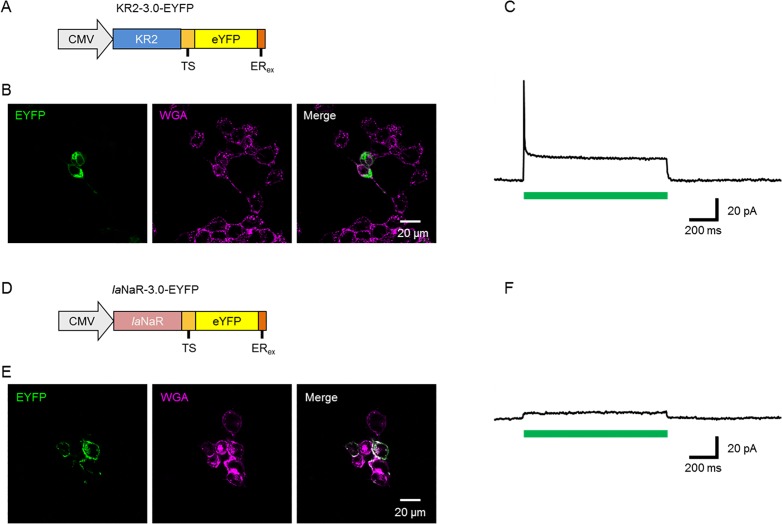
NaR expression in mammalian cells. (**A**) The expression plasmid, KR2-3.0-eYFP, consisted of the KR2 gene fused with a 20-amino-acid membrane-trafficking signal (TS), enhanced yellow fluorescent protein (eYFP), and 7-amino-acid ER-export signal (ER_ex_). (**B**) Expression of KR2-3.0-eYFP in the cultured ND7/23 cells: eYFP fluorescenc (left, green), wheat germ agglutinin (WGA) conjugated with Alexa Fluor 633 (middle, magenta) and the merge (right). (**C**) Typical photocurrent of KR2. The green bar indicates the period when green-yellow light (534–600 nm, 99 mW/mm^2^) was irradiated. (**D**) The expression plasmid, *Ia*NaR2-3.0-eYFP. (**E**) Expression of *Ia*NaR2-3.0-eYFP in the cultured ND7/23 cells: eYFP fluorescence (left, green), WGA (middle, magenta) and the merge (right). (**F**) Typical photocurrent of *Ia*NaR. The green bar indicates the period when green-yellow light (534–600 nm, 99 mW/mm^2^) was irradiated.

The photocurrent properties of *Ia*NaR were compared with KR2 by introducing a KR2 gene or *Ia*NaR gene fused with membrane-trafficking signal (TS), enhanced yellow fluorescent protein (eYFP), and ER-export signal (ER_ex_) [29] into cultured ND7/23 cells, hybrid cell lines derived from neonatal rat dorsal root ganglia neurons fused with mouse neuroblastoma [31]. The constructs were termed KR2-3.0-eYFP and *Ia*NaR-3.0-eYFP, respectively (Fig 3A and 3D). As shown in Fig 3B, the eYFP fluorescence was frequently found in the inclusion bodies in the KR2-expressing cells, suggesting the accumulation of misfolded/mistargeted proteins. However, every expressing cell responded to green-yellow light (534–600 nm, 99 mW/mm^2^) robustly with an outward photocurrent under a whole-cell clamp at a holding potential of 0 mV (Fig 3C). The KR2 photocurrent was rapidly activated to form a peak current (*I*_p_, 4.26 ± 0.97 pA/pF, n = 10), inactivated to form a steady-state current (*I*_ss_, 0.96 ± 0.20 pA/pF, n = 10) and deactivated to the baseline. As ND7/23 cells were live cell-stained with fluorescent WGA, the white merge indicated that the *Ia*NaR was mostly expressed in the membrane (Fig 3E). However, its photocurrent generated by green-yellow light (534–600 nm, 99 mWmm^-2^) was significantly smaller than that of KR2 for both *I*_p_ (0.22 ± 0.05 pA/pF, n = 10) and *I*_ss_ (0.19 ± 0.05 pA/pF, n = 10) (P < 0.005, Mann-Whitney *U*-test) (Fig 3F). Therefore, KR2 appears to be advantageous for optogenetic applications with larger photocurrent whereas *Ia*NaR is suitable for the exogenous expression with better membrane-targeting.

### Evaluation of chimeric NaRs

**Fig 4:**
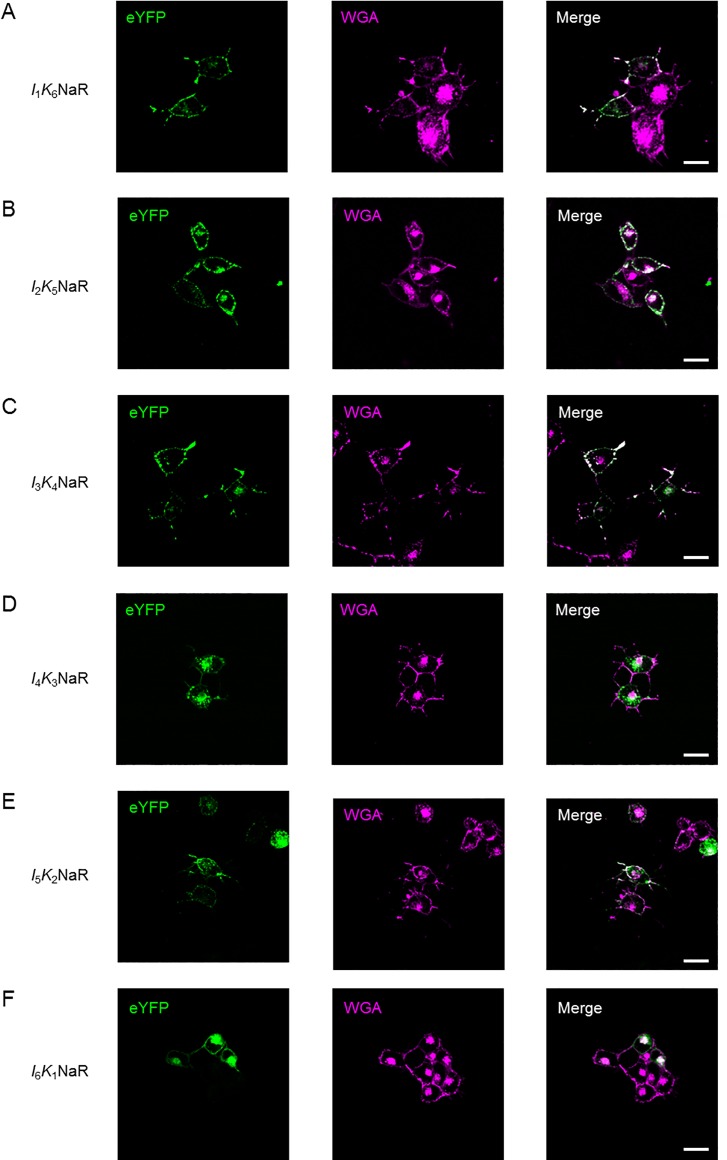
Expression of chimeric NaRs in ND7/23 cells. (**A**) *I*_1_*K*_6_NaR: eYFP fluorescence (left), fluorescent wheat germ agglutinin (WGA, middle) and the merge (right). (**B**)-(**F**) Similar to (A), but the expression pattern of *I*_2_*K*_5_NaR, *I*_3_*K*_4_NaR, *I*_4_*K*_3_NaR, *I*_5_*K*_2_NaR, *I*_6_*K*_1_NaR, respectively. Scale, 20 μm for each.

**Fig 5:**
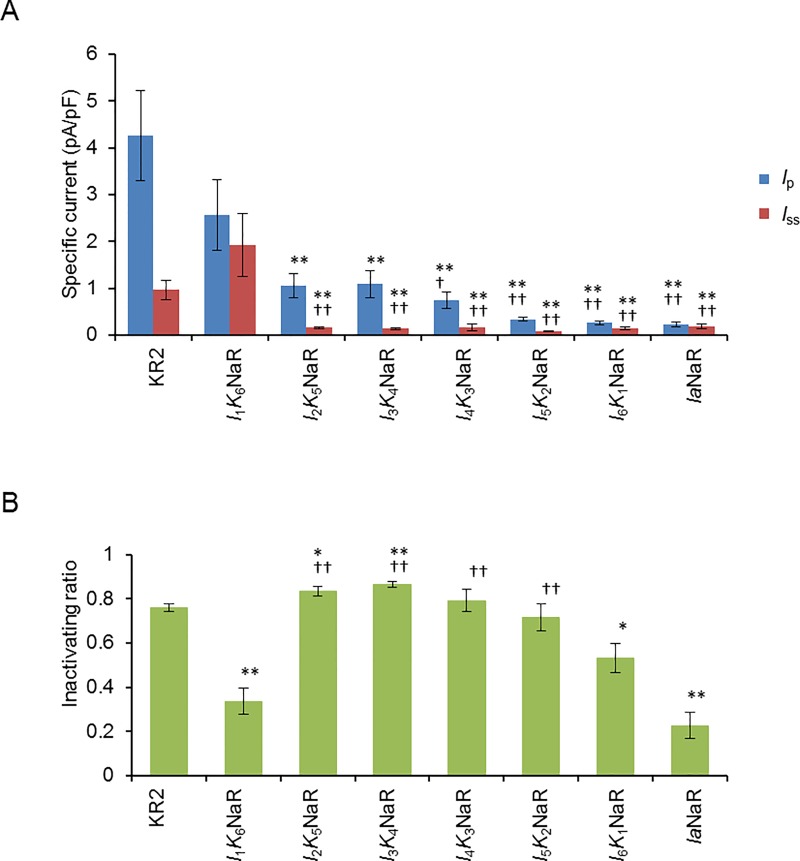
Photocurrent of chimeric NaRs. (**A**) The peak (*I*_p_, blue columns) and the steady-state (*I*_ss_, red columns) photocurrent at 0 mV holding potential in response to green-yellow light (534–600 nm, 99 mW/mm^2^) were compared among KR2 (n = 10), chimeric NaRs: *I*_1_*K*_6_NaR (n = 11), *I*_2_*K*_5_NaR (n = 10), *I*_3_*K*_4_NaR (n = 10), *I*_4_*K*_3_NaR (n = 10), *I*_5_*K*_2_NaR (n = 10), *I*_6_*K*_1_NaR (n = 9) and *Ia*NaR (n = 10). (**B**) Comparison of the inactivating ratio. The difference was significant when compared to KR2 (*, P < 0.05, **, P < 0.005, Mann-Whitney *U*-test) or *I*_1_*K*_6_NaR (^†^, P < 0.05, ^††^, P < 0.005, Mann-Whitney *U*-test).

The above results suggested the possibility that some chimeric NaRs between KR2 and *Ia*NaR may have the advantages of both NaRs, a larger photocurrent and improved membrane-targeting. To test this, six chimeric NaRs were made by replacing the N-terminal TMDs of KR2 with their counterparts of *Ia*NaR (Fig 1) on the assumption that the N-terminal domains of *Ia*NaR should be involved in the membrane-targeting. Each gene of chimeric NaR: *I*_1_*K*_6_NaR consisting of TMD1 from *Ia*NaR and TMD2-7 from KR2, *I*_2_*K*_5_NaR consisting of TMD1-2 from *Ia*NaR and TMD3-7 from KR2, *I*_3_*K*_4_NaR consisting of TMD1-3 from *Ia*NaR and TMD4-7 from KR2, *I*_4_*K*_3_NaR consisting of TMD1-4 from *Ia*NaR and TMD5-7 from KR2, *I*_5_*K*_2_NaR consisting of TMD1-5 from *Ia*NaR and TMD6-7 from KR2 and *I*_6_*K*_1_NaR consisting of TMD1-6 from *Ia*NaR and TMD7 from KR2, was fused with TS, eYFP and ER_ex_ and transfected to ND7/23 cells for the evaluation. Since TMD3 of *Ia*NaR and KR2 are completely identical to each other (Fig 1), *I*_2_*K*_5_NaR and *I*_3_*K*_4_NaR were the same in the amino-acid sequence but different as genes in the DNA sequence. As shown in Fig 4, the fluorescence expression of each chimeric NaR was similar to that of *Ia*NaR. Both the *I*_p_ and *I*_ss_ of *I*_1_*K*_6_NaR were significantly larger than those of the other chimeric NaRs as well as that of *Ia*NaR (Fig 5A). Therefore, *I*_1_*K*_6_NaR appeared to be a candidate chimeric NaR for optogenetic applications.

**Fig 6:**
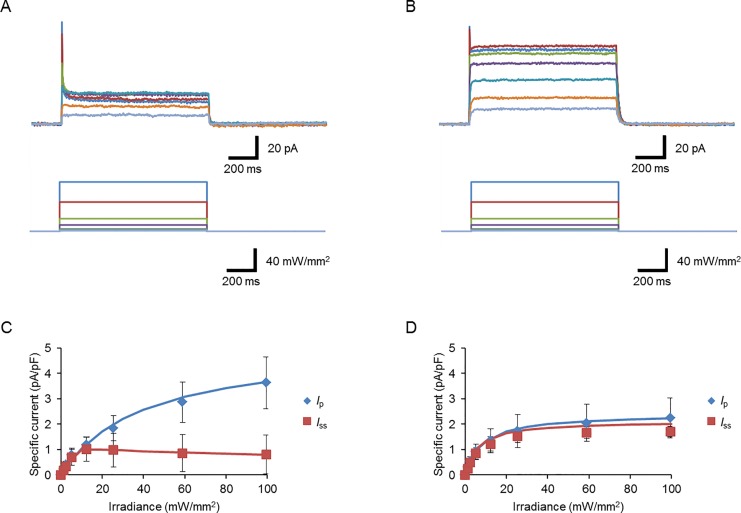
Photocurrent properties of *I*_1_*K*_6_NaR. (**A**) Sample photocurrents of KR2 (upper traces) at various powers of light (lower traces). (**B**) Sample photocurrents of *I*_1_*K*_6_NaR (upper traces) at various power of light (lower traces). (**C**) The amplitude of the peak (*I*_p_, blue diamonds) and steady-state (*I*_ss_, red squares) of the KR2 photocurrents as functions of irradiance (n = 8). The blue line is drawn according to the Michaelis-Menten relationship, *y* = 5.12*x* /(*x*+39.3). The red line is the Michaelis-Menten relationship from which the power-dependent current suppression component above a threshold of 10 mW/mm^2^ was subtracted, *y* = 2.8*x*/(*x*+18.7)– 2.3(*x*-10)/{(*x*-10)+38.1)}. (**D**) Similar to (C), but in the case of *I*_1_*K*_6_NaR photocurrent (n = 9). Each line is drawn according to the Michaelis-Menten relationship, *y* = 2.4*x* /(*x*+8.3) (blue) and, *y* = 2.1*x* /(*x*+6.2) (red), respectively.

Next, we compared the photocurrent properties of *I*_1_*K*_6_NaR with those of KR2 at the maximal irradiance (534–600 nm, 99 mW/mm^2^) and at a holding potential of 0 mV (Table 1). Similar to *Ia*NaR, the magnitude of inactivation of the *I*_1_*K*_6_NaR photocurrent was significantly smaller than KR2 (P < 0.005, Mann-Whitney *U*-test). It was also smaller than those of the other chimeric NaRs (Fig 5B). In the case of KR2, the *I*_p_ was monotonically increased with the increase of irradiance, whereas the *I*_ss_ had a tendency to reach the ceiling at around 20 mW/mm^2^ and was somewhat reduced with the increase of irradiance (Fig 6A and 6C). On the other hand, both the *I*_p_ and *I*_ss_ approached their ceilings with the increase of irradiance in the case of *I*_1_*K*_6_NaR (Fig 6B and 6D). The photocurrent-irradiance relationship is expected to follow Michaelis-Menten kinetics as the rhodopsin follows a single-photon photodynamism [32]. The apparent *K*_D_ of *I*_p_ was significantly smaller for *I*_1_*K*_6_NaR than for KR2 (Table 1, P < 0.05, Mann-Whitney *U*-test). Although the *I*_ss_ of *I*_1_*K*_6_NaR mostly followed the Michaelis-Menten relationship up to 99 mW/mm^2^, that of KR2 was well fitted to the Michaelis-Menten relationship from which a power-dependent current suppression component above a threshold of 10 mW/mm^2^ was subtracted. Therefore the *K*_D_ of *I*_ss_ was compared by fitting the data of irradiance between 0 and 12 mW/mm^2^ to the Michaelis-Menten relationship. The *K*_D_ of *I*_ss_ was significantly smaller for *I*_1_*K*_6_NaR than for KR2 (Table 1, P < 0.05, Mann-Whitney *U*-test).

**Table 1:** Photocurrent characteristics of KR2 and *I*_1_*K*_6_NaR.

	KR2	*I*_1_*K*_6_NaR
*I*_p_ [pA/pF]	4.26 ± 0.97 (n = 10)	**2.57 ± 0.75 (n = 11)
*I*_ss_ [pA/pF]	0.96 ± 0.20 (n = 10)	**1.92 ± 0.68 (n = 11)
Inactivation ratio	0.76 ± 0.02 (n = 10)	**0.34 ± 0.06 (n = 11)
*K*_D_ (*I*_p_) [mW/mm^2^]	39.3 ± 8.3 (n = 8)	*8.3 ± 1.1 (n = 9)
*K*_D_ (*I*_ss_) [mW/mm^2^]	18.7 ± 4.9 (n = 8)	*6.2 ± 1.4 (n = 9)
*S*_neg_ (*I*_p_) [10^3^mV^-1^]	4.03 ± 0.39 (n = 8)	2.45 ± 0.90 (n = 7)
*S*_neg_ (*I*_ss_) [10^3^mV^-1^]	0.43 ± 1.00 (n = 8)	**7.33 ± 2.13 (n = 7)

The photocurrents were responses to green-yellow light (534–600 nm) at 99 mW/mm^2^ except for *K*_D_ for which the power of light was variable.

*, P < 0.05

**, P < 0.005

Mann-Whitney *U*-test

**Fig 7:**
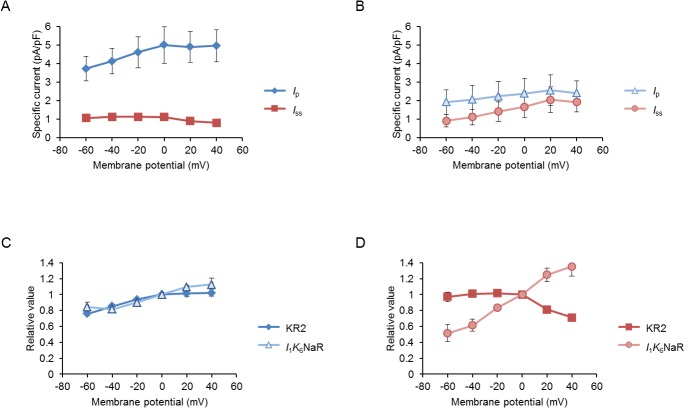
Sensitivity of *I*_1_*K*_6_NaR photocurrent to the membrane potential. (**A**) The current-voltage (*I-V*) relationship of the peak (*I*_p_, blue diamonds) and steady-state (*I*_ss_, red squares) of the KR2 photocurrents (n = 8). (**B**) The current-voltage (*I-V*) relationship of peak (*I*_p_, light blue triangles) and steady-state (*I*_ss_, light red circles) of *I*_1_*K*_6_NaR photocurrents (n = 7). (**C**) Each *I*_p_ of KR2 (blue diamond) and *I*_1_*K*_6_NaR (light blue triangle) was expressed as a relative value to that at 0 mV holding potential and plotted as a function of voltage. (**D**) Each *I*_ss_ of KR2 (red square) and *I*_1_*K*_6_NaR (light red circle) was expressed as a relative value to that at 0 mV holding potential and plotted as a function of voltage.

The *I*_1_*K*_6_NaR photocurrent differed from the KR2 photocurrent in the voltage sensitivity (Fig 7A and 7B). When normalized by the value at a holding potential of 0 mV, the *I*_p_ current-voltage (*I-V*) relationship was positively related to the holding potential for both NaRs (Fig 7C). Although the *I*_ss_ of KR2 was almost insensitive to the holding potential, particularly in the negative region, that of *I*_1_*K*_6_NaR was positively related to the holding potential (Fig 7D). To further test this, the slope of each normalized *I-V* relationship was calculated in the negative region of the holding potential (*S*_neg_). As shown in Table 1, the *S*_neg_ of *I*_1_*K*_6_NaR was significantly larger than that of KR2 for *I*_ss_ (P < 0.005, Mann-Whitney *U*-test) but not for *I*_p_.

**Fig 8:**
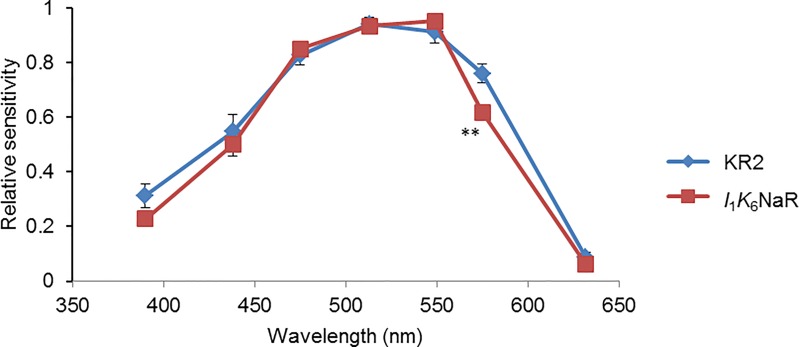
Action spectrum of *I*_1_*K*_6_NaR. For each wavelength the relative photocurrent amplitude (*I*_ss_) was divided by the irradiance, normalized to its maximum and averaged. The photocurrents of KR2 (blue diamonds, n = 9) and *I*_1_*K*_6_NaR (red squares, n = 12) did not show transient peaks because of the small magnitude of irradiance. **, P < 0.005, Mann-Whitney *U*-test.

The *I*_ss_ at each wavelengths (390, 438, 475, 513, 549, 575 and 632 nm at the center, respectively) was corrected by the irradiance (3.4, 3.0, 2.7, 2.7, 2.6, 2.8 and 2.8 mW/mm^2^, respectively) and its relative sensitivity to the maximum was averaged for KR2 and *I*_1_*K*_6_NaR and shown in Fig 8 as a function of the wavelength. Although the spectral sensitivity of the *I*_1_*K*_6_NaR photocurrent was almost similar to that of KR2, it was significantly lower in response to yellow light (575 nm) (P < 0.005, Mann-Whitney *U*-test).

### Light-dependent inhibition of neuronal activity

**Fig 9:**
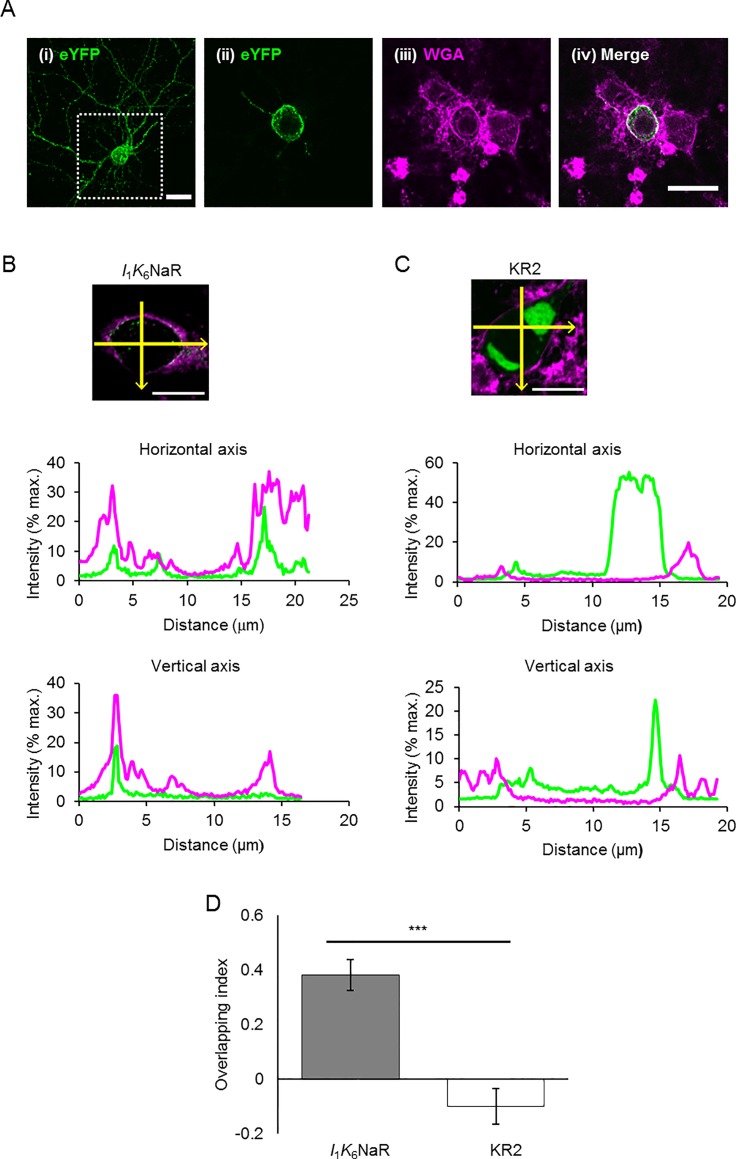
Quantitative evaluation of membrane targeting of NaRs. (**A**) Expression of eYFP-labeled *I*_1_*K*_6_NaR in the cultured cortical neuron. (**i**) Integrated confocal image of the neuron (Z-stack projection of 12 slices at every 0.46 μm in depth). (**ii**) eYFP image of a single slice shown in the dot-lined square in (i). (**iii**) Image of the fluorescent WGA of the same region. (**iv**) Merge of (ii) and (iii), indicating the expression of *I*_1_*K*_6_NaR in the neuronal membrane. (**B**) Profiling of the fluorescent intensity of an *I*_1_*K*_6_NaR-expressing neuron. The pixel values along the horizontal/vertical axes, which were delineated to pass the centroid of the cell (yellow), were plotted as functions of distance: eYFP fluorescence (green) and WGA fluorescence (magenta). The overlapping index was 0.68. (**C**) Profiling of the fluorescent intensity for a KR2-expressing neuron. The overlapping index was -0.21. (**D**) Comparison of the overlapping index between *I*_1_*K*_6_NaR (left) and KR2 (right). ***, P < 0.0005, Mann-Whitney *U*-test. Scales, 20 μm for (A) and 10 μm for (B) and (C).

Previously, the light-dependent activation of KR2 effectively silenced the neural activities both *in vitro* and *in vivo*. To test the possible application of *I*_1_*K*_6_NaR as an optogenetic silencer, the light-dependent changes of the membrane potential were investigated in cultured rat cortical neurons expressing *I*_1_*K*_6_NaR. As shown in Fig 9A, the *I*_1_*K*_6_NaR fluorescence was localized in the membrane when expressed in the cultured cortical neurons. Quantitative comparisons of the membrane targeting of the eYFP-labeled molecules were made between *I*_1_*K*_6_NaR and KR2 using primary cultured neurons from the cortex the plasma membrane of which was live cell-stained with a fluorescence marker, Alexa Fluor 633-labeled WGA. For each molecule, 10 neurons were randomly selected and the confocal images of their somas were quantitatively analyzed for localization of the fluorescence; the overlapping index of *I*_1_*K*_6_NaR was 0.38 ± 0.06 (n = 10) and positive for every neuron (range, 0.11~0.68) (Fig 9B). On the other hand, the overlapping index of KR2 fluorescence was -0.10 ± 0.06 (n = 10), negative in 7 of 10 neurons (range, -0.53~0.24) and significantly smaller than that of *I*_1_*K*_6_NaR (P < 0.0005, Mann-Whitney *U*-test) (Fig 9C and 9D).

**Fig 10:**
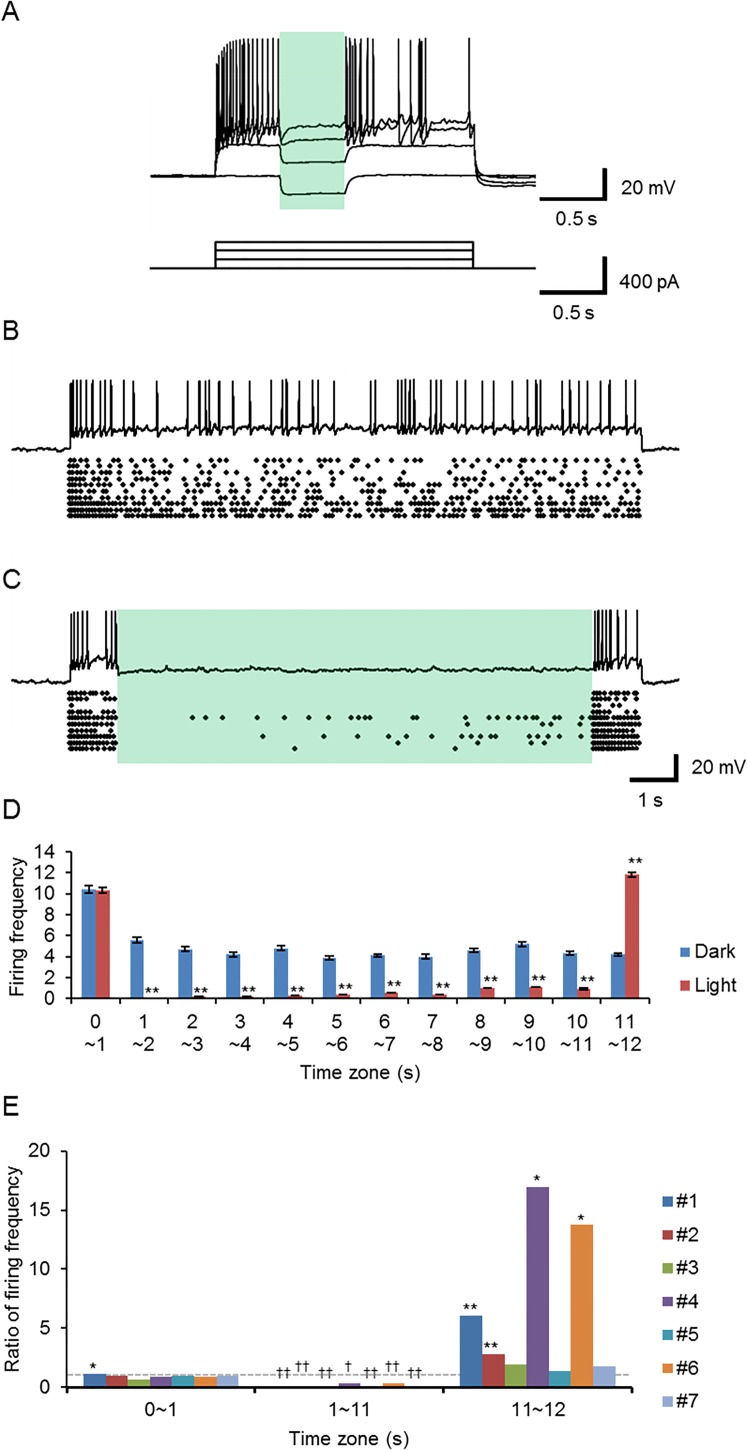
Light-dependent silencing of the neuronal activity. (**A**) Series of sample records of the membrane potentials of an *I*_1_*K*_6_NaR-expressing neuron under current clamp (top traces, RMP, -76 mV). The depolarization was made through current injection (bottom traces). The green light (549 nm, 2.6 mW/mm^2^) hyperpolarized the membrane potential and suppressed the generation of action potentials. (**B**) Generation of repetitive action poteintals in an *I*_1_*K*_6_NaR-expressing neuron under current clamp (top trace, RMP, -73 mV). The responses to 10 repetitive depolarizations are summarized as raster plots of the action potentials (bottom). (**C**) The activity of the same neuron was stably silenced during the green light irradiation (549 nm, 2.6 mW/mm^2^) for 10 s (top trace, RMP, -73 mV). The responses to 10 repetitive depolarizations are summarized as raster plots of action potentials (bottom). (**D**) The firing frequencies during time zones of 1 s each. The effects of green light irradiation (time zones from 2~11 s) were compared for the same neuron shown in (A) and (B); without (‘Dark’ protocol, blue columns) and with light (‘Light’ protocol, red columns). Note the significant increase of firing frequency at the off period (time zone 11~12 s). (**E**) Ratios of firing frequency of ‘Light’ to ‘Dark’ protocol during time zone 0~1 s (before irradiation), 1~11 s (during irradiation), 11~12 s (after irradiation), respectively for each neuron #1~7 expressing *I*_1_*K*_6_NaR (experimental order). The gray dotted line indicate that the ratio = 1. *, P < 0.05; **, P < 0.005; Mann-Whitney *U*-test applied to the set of records. ^†^, P < 0.05; ^††^, P < 0.005; Wilcoxon signed rank test applied to the firing frequency for time zone, 1~2, 2~3, 3~4, 4~5, 5~6, 6~7, 7~8, 8~9, 9~10 and 10~11, respectively.

Under current clamp, the resting membrane potentials (RMPs) were between -51 and -83 mV (-64 ± 4 mV, n = 11) and hyperpolarized by 8–24 mV (-15 ± 5 mV, n = 11) with light (549 nm, 2.6 mW/mm^2^). Although action potentials were repetitively generated by the depolarization through current injection, they were hardly evoked during irradiation of light (Fig 10A). In every experiment (n = 11), the action potentials, evoked once, were reversibly inhibited during irradiation (549 nm, 2.6 mW/mm^2^). The light-induced inhibition of firing was quite stable and remained effective for as long as 10 s (Fig 10B–10D). In summary of 7 similar experiments (Fig 10E), the firing frequency was significantly smaller throughout the irradiation period (10 s, 549 nm, 2.6 mW/mm^2^) than the corresponding period in the darkness (P < 0.05, Wilcoxon signed rank test). The firing frequency was often increased when the irradiation was turned off (rebound potentiation, Fig 10C and 10D). Indeed, a significant increase of firing frequency was observed in 4 of 7 similar experiments (Fig 10E).

## Discussion

This study addressed the question whether a new Na^+^-pump rhodopsin from *Indibacter alkaliphilus* (*Ia*NaR) could serve as an optogenetic silencer with negligible disturbance of the ionic milieu both inside and outside of the membrane [27]. The ion-transport assay with *E*. *coli* cells showed that *Ia*NaR outwardly transports Na^+^ when the internal solution contained Na^+^, but H^+^ when all the Na^+^ was replaced by K^+^. This ion-transporting property and selectivity is identical to KR2, the first reported NaR [26], and NaR from *Dokdonia sp*. PRO95 [33]. λ_max_ of *Ia*NaR (528 nm) is also similar to KR2 (524 nm) [26] and NaR from *Dokdonia sp*. PRO95. (523 nm) [33]. Therefore, the fundamental properties of *Ia*NaR including the ion-transporting function and the structure around retinal are similar of those of previously reported NaRs. When *Ia*NaR was expressed in the mammalian cells such as ND7/23, its photocurrent was very small even under the maximal power of green-yellow light (534–600 nm, 99 mW/mm^2^). However, it appeared to be better targeted to the plasma membrane than KR2.

The eYFP-labeled KR2 molecules frequently accumulated in large intracellular compartments even after the gene was fused with a TS and an ER_ex_ which facilitates the plasma membrane expression of the transcribed rhodopsins [29]. The exogenously expressed membrane proteins are often misfolded resulting in the ER retention or degradation with a reduction of functional membrane proteins at their correct sites [34]. The accumulation of misfolded proteins also causes ER stress, which can lead to cell death if remains unmitigated [35,36]. It is possible that *Ia*NaR could provide conformations that help the rhodopsin to become folded correctly and to be targeted to the plasma membrane with minimal ER stress. Indeed, the chimera rhodopsins consisting of the N-terminal TMDs from *Ia*NaR and the C-terminal TMDs of KR2 appeared to be well targeted to the plasma membrane rather than in the intracellular compartments of ND7/23 cells. Among the chimeras, *I*_1_*K*_6_NaR consisting of TMD1 from *Ia*NaR and TMD2-7 from KR2 generated *I*_p_ and *I*_ss_ as large as KR2. However, it remains puzzling that the magnitude of the photocurrent of *Ia*NaR and some of its chimeric derivatives was similar to or lower than that of KR2 even with the improved membrane targeting. The possibility that the ion-transporting efficiency of *Ia*NaR and its derivatives is lower than that of KR2 should be investigated in future. Alternatively, the membrane-targeted molecules may be relatively rapidly internalized and/or denatured in the case of *Ia*NaR and its derivatives. Further modification of molecules, focusing on the N-terminal extracellular segment and TM1, might create an NaR with larger photocurrent amplitude and better membrane targeting [37–39].

Similar to KR2, the ion-transport assay with *E*. *coli* cells showed that *I*_*1*_*K*_*6*_NaR outwardly transports Na^+^ when the internal solution contained Na^+^, but H^+^ when all Na^+^ was replaced by K^+^ (S1 Fig). However, the photocurrent properties of *I*_1_*K*_6_NaR were somewhat different from those of KR2. Although the difference was insignificant, the *I*_ss_ of *I*_1_*K*_6_NaR was relatively large but with a significantly smaller inactivation ratio. The *I*_1_*K*_6_NaR was significantly more sensitive to light (534–600 nm) than KR2 in both *I*_p_ and *I*_ss_. As the spectral sensitivity of *I*_1_*K*_6_NaR was similar to KR2, the different sensitivity may be attributed to the probability of conformational changes at the molecular level upon light absorption. Although KR2 photocurrent was almost insensitive to voltage in both *I*_p_ and *I*_ss_ as reported previously [27], the *I*_ss_ of *I*_1_*K*_6_NaR was positively related to the voltage. These photocurrent properties as well as its membrane-targeting trait would suggest that *I*_1_*K*_6_NaR could become one of the optogenetic NaRs for long-term silencing of neural activities with relatively low power of light.

Upon the cessation of light exposure, the rapid recovery of the membrane potential was often accompanied by the rebound potentiation with the possible changes in the membrane properties during hyperpolarization: the reduced inactivation of voltage-dependent Na^+^ channels, the deactivation of voltage-dependent K^+^ channels, and the activation of HCN channels (*I*_h_). Indeed, the NaR-dependent neural silencing was often accompanied by an increase in the firing frequency upon termination of light pulse. Although NpHR and AR3/ArchT are widely-used optogenetic tools for neural silencing, they still have much room for improvement. For example, several papers reported that NpHR triggers artificial neural spiking relatively long after turning-off the light [40–42]. As intracellular Cl^-^ accumulation would inevitably accompany the positive shift of the Cl^-^-equilibrium potential, it is reasonable to assume that the Cl^-^ pumping activity of NpHR enhances the magnitude and duration of the rebound potentiation. In addition, this Cl^-^ accumulation may not be a trivial problem in some cases, such as *in vivo* optogenetic analysis of mammalian neurons during developmental stages. In adult mammals, the intracellular Cl^-^ concentration is maintained at a low level by several transporters, such as KCC2. However, in developmental stages, when the Cl^-^ extrusion system is still immature, the intracellular Cl^-^ concentration is relatively high with depolarized Cl^-^ equilibrium potential [43,44]. Similarly, it is assumed that the intracellular Cl^-^ accumulation by the activity of the light-driven Cl^-^ pump further enhances the magnitude and duration of the rebound potentiation with repetitive firing, and makes it difficult to infer the functions of target neurons and neural circuits in the behavioral response [45]. In the case of AR3/ArchT, the light-dependent change of local pH around the neurons can activate the proton-gated cation channel such as ASIC (acid sensing ion channel) and cause unintentional rebound potentiation [46]. The AR3/ArchT-dependent intracellular alkalization may lead to the unintentional release of neurotransmitter through triggering Ca^2+^ influx [47]. Similar cautions will be necessary in the case of light-gated Cl^-^/anion channels [12–15] as the direction of membrane potential change is dependent on the equilibrium potential of Cl^-^ that can be affected by many factors such as development, localization and disease [18–25]. Distinct from the Cl^-^/anion channel rhodopsins, the hyperpolarization of the membrane potential is always expected for any NaR because of the unidirectional transport of Na^+^. Indeed the *I*_1_*K*_6_NaR-expressing cortical neurons were stably silenced by green light irradiation for a rather long duration. In addition, the artefactual change of the pH balance is expected to be almost negligible as the H^+^-transporting rate is estimated to be less than 3% those of Na^+^. This estimation based on the higher Na^+^ activity than H^+^ (320,000-fold) inside a mammalian cell in a physiological condition such as 10 mM Na^+^ and pH 7.3, whereas the rate constant of H^+^ uptake was larger than that of Na^+^ uptake with a ratio of 8,000–9000 [48]. Furthermore, one of the marked advantages of *I*_1_*K*_6_NaR is its *I-V* relationship: the photocurrent is smaller at the resting membrane potential while larger at the depolarized potential. Under the current-clamp condition, the outward transport of Na^+^ by the irradiated *I*_1_*K*_6_NaR is increased by the depolarization before generation of the action potential. As a result, the action potential is inhibited and the membrane potential is hyperpolarized, which on the other hand, decreases the outward Na^+^ flow. Therefore, with its rapid kinetics, the photoactivation of *I*_1_*K*_6_NaR could become an ideal optogenetic silencer that specifically suppresses the generation of action potentials with less hyperpolarization of the neuronal membrane potential than KR2. Since the rebound potentiation is dependent on the magnitude of the hyperpolarization and on the duration, its magnitude is expected to be less for *I*_1_*K*_6_NaR.

### Conclusions

We found that one of the chimeras between KR2 and *Ia*NaR, named *I*_1_*K*_6_NaR, exhibited some improvements in membrane targeting and photocurrent properties over native NaRs. The *I*_1_*K*_6_NaR would be a potential candidate of the effective optogenetic neural silencer with minimal influence on the ionic/pH balance.

## Supporting Information

S1 Fig Ion-transport activity of *I*_1_*K*_6_NaR. (**a**-**c**) Light-induced pH changes upon light-illumination (>500 nm, indicated by yellow lines) on the *E*. *coli* cells expressing *I*_1_*K*_6_NaR in 100 mM NaCl (**a**), 100 mM Na_2_SO_4_ (**b**) and 100 mM KCl (**c**) without (blue lines) and with CCCP (green lines).
(PDF)
