# Supplementary material for: A Chimera Na+-Pump Rhodopsin as an Effective Optogenetic Silencer
Source: PLoS One. 2016 Nov 18;11(11):e0166820. doi: 10.1371/journal.pone.0166820 (PMC5115807; doi:10.1371/journal.pone.0166820)
Supplement: S1 Fig — (a-c) Light-induced pH changes upon light-illumination (>500 nm, indicated by yellow lines) on the E. coli cells expressing I1K6NaR in 100 mM NaCl (a), 100 mM Na2SO4 (b) and 100 mM KCl (c) without (blue lines) and with CCCP (green lines). (PDF) [file pone.0166820.s001.pdf]

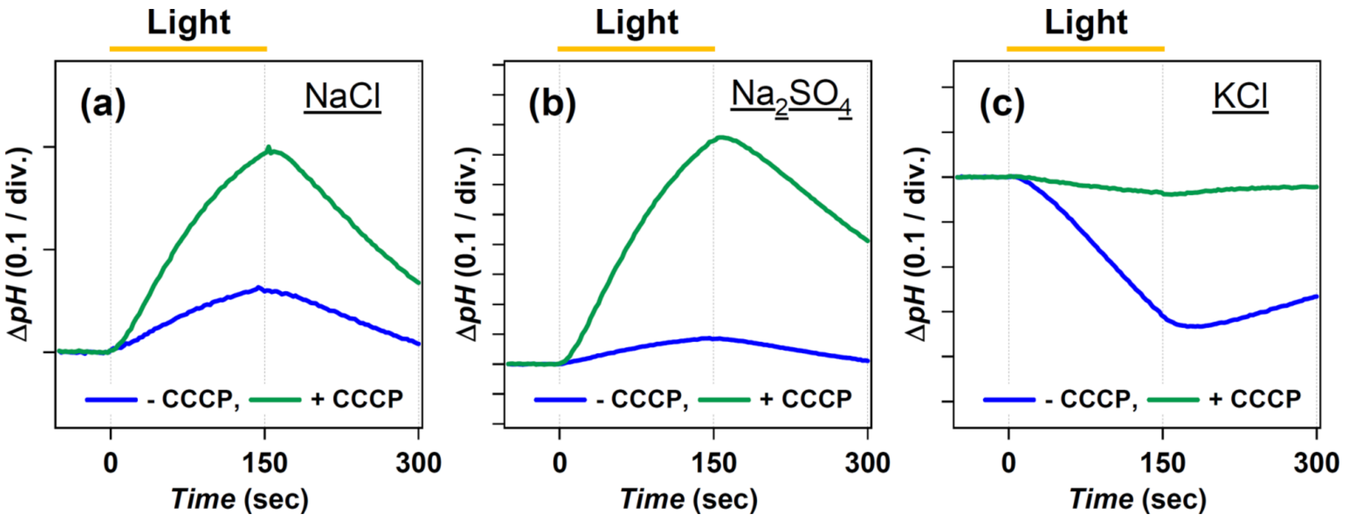

S1 Fig. Ion-transport activity of I_1K_6NaR. (**a-c**) Light-induced pH changes upon light-illumination (>500 nm, indicated by yellow lines) on the *E. coli* cells expressing I_1K_6NaR in 100 mM NaCl (**a**), 100 mM Na_2SO_4 (**b**) and 100 mM KCl (**c**) without (blue lines) and with CCCP (green lines).
